# How an Immune-Factor-Based Formulation of Micro-Immunotherapy Could Interfere with the Physiological Processes Involved in the Atopic March

**DOI:** 10.3390/ijms24021483

**Published:** 2023-01-12

**Authors:** Camille Jacques, Ilaria Floris

**Affiliations:** Preclinical Research Department, Labo’Life France, Pescalis-Les Magnys, 79320 Moncoutant sur Sevre, France

**Keywords:** atopic march, allergen immunotherapy, cytokines, ultra-low doses, micro-immunotherapy, atopic dermatitis, allergic rhinitis, allergic asthma

## Abstract

Allergic diseases consist of improper inflammatory reactions to antigens and are currently an important healthcare concern, especially considering their increasing worldwide development in recent decades. The “atopic march” defines the paradigm of allergic diseases occurring in chronological order and displaying specific spatial manifestations, as they usually start as atopic dermatitis (AD) and food allergies during infancy and progressively evolve into allergic asthma (AA) and allergic rhinitis (AR) or rhino-conjunctivitis in childhood. Many immune cell subtypes and inflammatory factors are involved in these hypersensitivity reactions. In particular, the T helpers 2 (Th2) subset, through its cytokine signatures made of interleukins (ILs), such as IL-4, IL-5, IL-10, and IL-13, as well as mast cells and their related histamine pathways, contribute greatly to the perpetuation and evolution of the atopic march. By providing low doses (LD) and ultra-low doses (ULD) of ILs and immune factors to the body, micro-immunotherapy (MI) constitutes an interesting therapeutic strategy for the management of the atopic march and its symptoms. One of the aims of this review is to shed light on the current concept of the atopic march and the underlying immune reactions occurring during the IgE-mediated responses. Moreover, the different classes of traditional and innovative treatments employed in allergic diseases will also be discussed, with a special emphasis on the potential benefits of the MI medicine 2LALERG^®^ formulation in this context.

## 1. The Atopic March Paradigm

**Figure 1:**
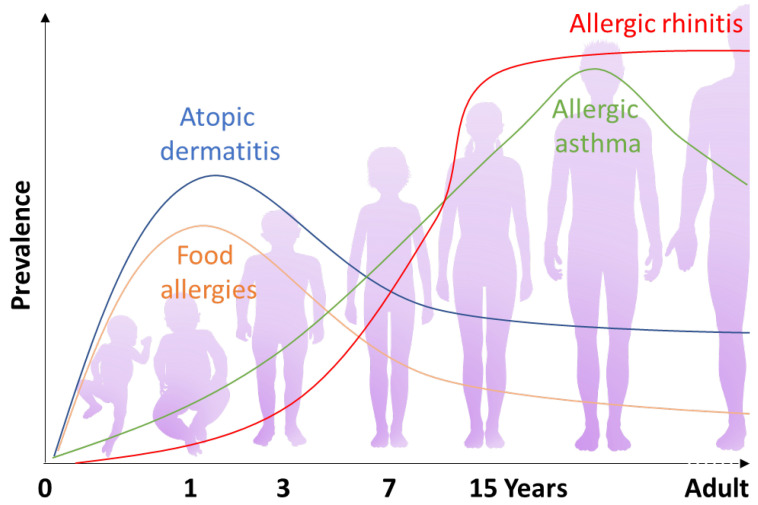
Schematic representation of the prevalence of the different conditions that constitute the atopic march over time. Atopic dermatitis and IgE-mediated food allergies are generally the first to develop early on during infancy. Allergic asthma and allergic rhinitis often follow, appearing later on during childhood.

Allergic diseases have become an increasing problem in civilization in recent decades, as allergic symptoms currently affect 150 million Europeans, according to the European academy of allergology and clinical immunology (EAACI) [1]. Coca and Cooke were the first to introduce the term “atopy” and classify the phenomenon of hypersensitiveness in 1923 [2]. Atopy defines the inappropriate immune reaction to allergic stimuli, mediated by high immunoglobulin (Ig) E levels, ultimately leading to clinical manifestations at various localizations within the body. In general, a predisposition to allergy development is shown in atopic individuals, and its manifestation is often seen as a consequence of a characteristic chronological pattern, the so-called “atopic march” (Figure 1). This step-by-step process usually evolves from atopic dermatitis (AD) (also known as hay fever) and food allergies apparition early on during infancy to the emergence of allergic asthma (AA) and allergic rhinitis (AR) later on in childhood. This gradual evolution of the allergic features manifests in parts of the body such as the skin, the digestive tract, and the airway-related axis, which are all anatomical surfaces consisting of epithelial cells continuously exposed to environmental threats [3]. Consequently, it is understood that a combination of (i) a dysregulation in the epithelial barrier favoring allergen penetration and (ii) an unbalanced and overreactive immunological response against allergens caused by activated T helpers 2 (Th2) cells and aberrant regulatory T-cells (Treg) are likely the two main causes responsible for the onset of the atopic march and the allergic manifestations in general [4].

### 1.1. The First Step of the Atopic March: Atopic Dermatitis

The first manifestation of the atopic march appears in the form of AD, a chronic, recurrent inflammatory skin affection characterized by intractable pruritus and whose etiopathogenesis is still not completely elucidated. The prevalence of this systemic disease is approximately 15–20% in children and between 1 and 3% in adults [5]. The chronic nature of AD is revealed by the fact that its complete resolution before the age of 7 only happened in less than 50% of AD patients, while symptom remission during adulthood occurs in 60% of cases [6]. The symptoms of AD include dry and scaly skin, eczema lesions, and chronic itching. Not only do the repercussions of these manifestations highly impact the quality of life of patients suffering from this condition, but it is also worth noticing that the disrupted skin barrier constitutes an opportunity for secondary infections [7].

From a cellular standpoint, the disease is initiated by contact between external allergens and the epithelial cells (keratinocytes) of the damaged skin. Consequently, these epithelial cells are triggered to secrete various factors, such as thymic stromal lymphopoietin (TSLP), which act as activators of the Th2′s responses. The next step in disease progression is the production of non-specific IgE and skin infiltration with T-cells, eosinophils, macrophages, mast cells, and type 2 innate lymphoid cells (ILC2), all of them producing pro-inflammatory cytokines that contribute to local inflammation perpetuation [8]. A plethora of immune factors and pro-inflammatory cytokines are involved in AD allergic reactions, such as interleukin (IL)-1, IL-6, or tumor necrosis factor α (TNF-α), which are released by cells such as inflammatory dendritic epidermal cells. Furthermore, not only does the immune dysregulation characterizing AD occur locally, but it also spreads at a systemic level. Some of these disturbances include increased circulating IgE and sensitization to allergens, elevated Th2-type cytokine expression, or increased numbers of T-cells expressing the homing skin receptor cutaneous lymphocyte-associated antigen (CLA) [9]. Moreover, particular T-cell subsets, characterized by immunosuppressive properties, are also found in AD patients at systemic levels, including CD4^+^, CD25^+^, FoxP3^+^, and Treg, which can help in the process of healing and tissue repair [10].

Finally, the course of AD is significantly related to food sensitivity, especially in children with hen’s egg sensitization who show a longer persistence of the condition [11]. Food sensitization is defined as having detectable levels of food-specific IgE, which can be a precursor to the development of clinical food allergies. In short, this process is a mistaken identification of food antigen(s) as potential pathogen(s). The clinical manifestations can be mild, moderate, or severe (or cause death) and can involve almost every organ system, including the skin, the respiratory tract, and the gastrointestinal tract, as well as the cardiovascular and neurological systems [12,13]. From an epidemiologic standpoint, persistent IgE-positive AD is linked and leads to a higher risk of developing AA and AR, as confirmed in several studies [14].

### 1.2. The Progression of the Atopic March: Allergic Rhinitis and Allergic Asthma

The progression of the atopic march over time results in the development of two strongly associated chronic inflammatory conditions, AA and AR, usually both designated under the broad denomination of allergic airway diseases (AAD) [15], and both affect the nasal and/or lung epithelium. While the mechanism has not been completely elucidated, it is supposed that allergens can directly compromise the nasal/lung epithelial barrier, thus, initiating innate immune responses through the release of alarmins such as IL-33, TSLP, or IL-25. Alarmins can, in turn, activate ILC2 cells, which induce the rapid in situ production of type 2 cytokines (IL-5, IL-13, and IL-4) and the consequent IgE-mediated mucosal inflammation [16,17]. The allergic response begins with a sensitization phase, which leads to the formation of a pool of memory-allergen-specific Th2 and B cells. During this phase, the patients do not experience clinical symptoms. The subsequent allergen exposure activates basophils, macrophages, and mast cells in situ, triggering the release of allergic mediators, such as histamines, and manifesting the classical acute symptoms. This process is characterized by mucus hypersecretion, airway inflammation, hyperresponsiveness, and airway remodeling. The subsequent and persistent cytokine production can cause an inflammatory infiltrate, with eosinophil recruitment and chronic inflammatory situations [18].

Epidemiology data estimate the prevalence of AA in Europe at 8.2% in adults and 9.4% in children [19]. Additionally, the prevalence of subjects with clinically confirmable AR ranged from 17% in Italy to 29% in Belgium, according to a cross-sectional population-based survey conducted amongst European adults [20]. In pediatric populations, surveys conducted all over the world from the international study of asthma and allergies in childhood (ISAAC) reported AR prevalence variations ranging from 0.8% to 14.9% in 6–7-year-olds and from 1.4% to 39.7% in 13–14-year-olds, the lowest prevalence being found in parts of Eastern Europe and South and Central Asia [21].

From a clinical perspective, AR is a non-infectious inflammatory disease affecting the nasal mucosa, mediated by IgE, and triggered by one or more environmental allergen(s) in a genetically susceptible population. AR is frequently associated with asthma [6], usually persists throughout life, and is largely classified as perennial, episodic, or seasonal, depending on the temporal pattern of the symptoms. Seasonal causes include aeroallergen pollen from grass, weeds, or trees, whereas, in perennial conditions, environmental contaminants such as animal dander, house dust mites, fungus spores, feathers, as well as cockroach proteins or molds are commonly reported as the causes of the main symptoms. The frequency of AR varies and can either be described as intermittent (less than 4 days/week or less than 4 weeks/year), or persistent (more than 4 days/week or 4 weeks/year) [22]. The clinical manifestations are multiple, vary in duration/severity, and include typical paroxysmal sneezing, runny/itchy nose, clear nasal discharge/congestion, and airflow obstruction [23]. While this disease is often trivialized, its symptoms’ scale ranges from mild to severe and can, unfortunately, largely impact the patient’s quality of life, performance, and even attendance at school/work [24].

### 1.3. Genetic and Environmental Factors Are Involved in the Etiopathogenesis of the Atopic March

The etiopathogenesis of the atopic march is multifactorial, and although there is no single gene responsible for its onset, genetic susceptibility and immunity—as well as environmental factors—were shown to be involved in its progression. One of the main risk factors is family history. In this regard, it was reported that maternal atopy and allergic comorbidities are associated with a higher risk of developing allergies in children [25]. Due to large-scale genome-wide association studies, multiple susceptibility loci were identified [26]. While the point of this manuscript is not to discuss the genetic alterations involved in the onset of the atopic march, those affecting the main immunologic factors and cytokines of interest are thoroughly discussed in the following section.

#### 1.3.1. Genetic Factors Involved in the Etiopathogenesis of the Atopic March

The polymorphisms of three *IL-1* gene complex genes, namely, *IL1A* (+4845G>T), *IL1B* (-511 degrees C>T), and *IL1RN* (variable number of tandem repeats; IVS2, 86 bp, duplicates 2 to 5) were compared between AR patients and non-asthmatic individuals [27]. The genotype distribution was reported to differ significantly between these groups, thus, highlighting the impact of the genetic background of this cytokine during the AR onset. Even though there are several pieces of evidence linking *IL1* to AR, the direct genetic association of *IL1* with AD has been documented less and is controversial. Indeed, while one study reported that IL-1 levels were elevated more in the *stratum corneum* of AD patients bearing mutations for the skin-barrier-related gene *filaggrin*, and this expression profile was also reported in a murine homolog of filaggrin deficiency [28]; a negative association was also found between *IL1B* and AD disease [29].

The results from a study conducted on 89 Iranian children with AD and 139 healthy controls reported a significant increase in the frequency of the G allele and GG genotype at position −174 of *IL6* [30]. Furthermore, a meta-analysis including 11 studies performed on the general population revealed a statistically significant association between *IL6* rs1800795 polymorphism and the risk of developing AA and AR [31].

Interleukin-4 plays a key role in AR pathogenesis. A recent meta-analysis reporting the results from studies mainly conducted in Asian countries not only suggested a significant correlation between *IL-4* rs2243250 single-nucleotide polymorphism and susceptibility to AR, but also that allele T and genotype TT could increase the risk of AR [32]. Furthermore, it was shown that *IL4*-hemizygosity differentially affected ovalbumin (OVA)-specific IgE antibody production as immunized *IL4*-hemizygous mice generated fewer IgE antibody-secreting cells than IL-4^+/+^ mice, resulting in lower levels of IgE bound to basophils and mast cell membranes, as well as in the circulation [33]. Interestingly, a multi-stage genome-wide association study conducted on infantile eczema followed by childhood AA, including 2428 cases and 17,034 controls, reported significant susceptibility loci for this characteristic pattern of allergic disease, including *IL4/KIF3A* (5q31.1) [34]. Another “omics” etiology study conducted in the context of ancestry variations also reported the pathogenic involvement of *IL4*, *IL5*, *IL10*, *IL13,* and *IL4R* genes in the atopic march [35].

The heterodimeric subunits of the IL-12 cytokine are encoded by two distinct genes, *IL12A* and *IL12B*, and as this cytokine is implicated in the allergen-induced inflammation through CD4^+^ T-cells’ polarization into Th1 cells, its targeting could be of great value in AR treatment. Interestingly, an Iranian case–control study, including 130 AR patients and the same number of healthy controls, found that the *IL12B* gene could be a potential biomarker for AR predisposition, as the GC genotype of rs6887695 G>C was associated with susceptibility to AR in comparison with the GG genotype [36]. Moreover, in the context of AD, it was suggested that individuals bearing the *IL12RB1* promoter −111T/T polymorphism displayed a reduced IL-12Rβ1 expression that may have led to increased Th2 cytokine production in the skin and contributed to the development of AD and other subsequent allergic diseases.

Furthermore, it was reported that house dust mite (HDM) sensitization is associated with asthma diagnosis during the lifetime, as well as with current asthma and bronchial hyperresponsiveness in Korean children, and the genetic polymorphism of *TFN-α* rs1800629 was found to modify and interact with these associations [37].

Genetic susceptibility to AD in diverse, non-European ethnic groups was studied, and the results revealed that mutations or allele repeats were found in genes associated with immune regulation, especially those related to innate host defenses, T-cell function, and autoimmunity [38]. For instance, the genes reported in this study were *human β-defensin-1 (DEFB1)*, *IL-4, IL-4Rα, IL-4R, IL-5, IL-12B, IL-12RB, IL-13, IL-13Rα1, Fc fragment of IgE receptor Ia (FCER1A), TNF receptor superfamily member 6b (TNFRSF6B),* and *human leukocyte antigen-DRB/DQA1* (*HLA-DRB/HLA-DQA1)*. In line with these results, complete reviews also put together case–control studies and genome-wide association studies, highlighting the link between HLA-II and respiratory allergic diseases [39] and between human leukocyte antigen type II (HLA-II) and food allergies, conditions which are also implicated into the atopic march process [40].

Altogether, this evidence nicely depicts that genetic predispositions related to the cytokine background of an individual could be linked with atopic march susceptibility.

#### 1.3.2. Perinatal Environmental Factors Involved in the Etiopathogenesis of the Atopic March

**Figure 2:**
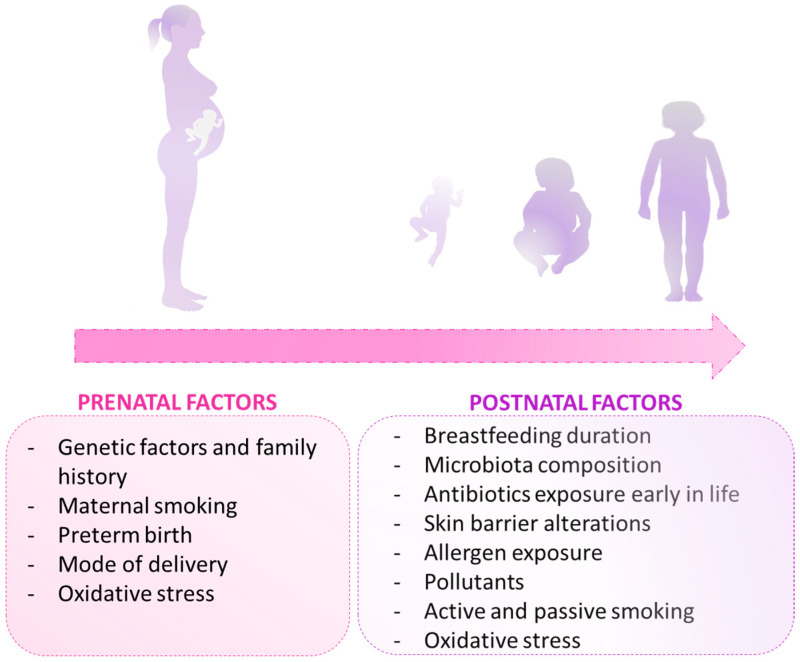
A non-exhaustive list of the main perinatal factors involved in the atopic march in different stages of life (pre- and post-birth). Genetic and environmental factors are implicated in the onset and progression of the atopic march. The environmental factors at the fetal stage (in utero), at birth, or after birth can affect the individual predisposition to allergic diseases. Some genetic backgrounds related to the cytokines and immune factors of interest in this review are discussed in Section 1.3.1.

With regard to perinatal environmental factors, the main predicted risk factors are cited in Figure 2 and are grouped according to the specific life stages (from prenatal to post-natal period). Maternal (or parental) smoking exposure during the intrauterine period increases the risk of childhood asthma susceptibility [41]. Preterm birth is recognized as a possible risk factor for atopic diseases; a recent long-term observational study demonstrated that more than 40% of children prematurely born (with gestational age <32 weeks) developed atopic manifestations during the first 5 years of life [42]. Moreover, the mode of delivery was also revealed to influence the occurrence of asthma and allergy in later life; it was observed that cesarean delivery greatly increases the risk of childhood asthma. This is because the mode of birth strongly influences the composition of the microbiota and, in turn, the immune system [43]. Indeed, children born by vaginal delivery showed a higher flora/microbiological diversity with a predominance of *Lactobacilli* compared to children born by cesarean section, who showed gut dysbiosis. During the perinatal period, one of the cited predicted factors associated with a reduced risk of developing atopic march is breastfeeding duration. Breast milk is not only considered to provide optimal nutrition during the first six months of life, but it is also known as an immunologically living biological substance containing molecules that contribute to infant growth and promote the development of host defense mechanisms. According to a recently published study (based on a questionnaire including approximately 10,700 participants), a longer breastfeeding duration was associated with a reduced odds ratio of childhood asthma and allergic diseases [44]. Other factors, such as the overuse of antibiotics, especially during the first few years of life, can alter the microbiota composition and increase the risk of allergic disease development [45,46].

In parallel with perinatal-related factors, an impairment in the skin barrier is also a predisposing factor for AD and subsequent asthma due to the facilitation of allergens and microorganisms’ entry, leading to further allergic sensitization [47]. Moreover, exposure to allergens and pollutants such as nitrogen dioxide, nitric oxide, sulfur dioxide, carbon monoxide, and carbon dioxide, as well as active/passive smoking exposure, represent additional risks that can predispose to allergic diseases [48,49]. Finally, increased oxidative stress (during both pre- and post-natal periods) was identified as another risk factor contributing to the progression, persistence, and exacerbation of allergic inflammation associated with the multifactorial etiopathogenesis of the atopic march [50].

### 1.4. How Can We Stop the Atopic March?

#### 1.4.1. Current Treatments against AR and AD

Regarding the tremendous increase in both the prevalence and the harshness of these pathologies over the past few years, managing their symptoms through appropriate treatments is now considered a medical, economic, and global public health concern. The severity and persistence of the symptoms are two major parameters that orient healthcare professionals toward the best-suited therapeutic strategy for each individual. The different options are often chosen from the following or a combination thereof: (i) prevention measures and protective factors, (ii) symptomatic treatments, (iii) allergen-specific immunotherapy (ASIT), and/or (iv) targeted therapies.

##### Prevention Measures and Protective Factors

Since they do not require any medication, systematic daily measures aiming at reducing allergen exposure sound appealing initially. However, no significant alleviation in symptoms or improvement in quality of life in patients with AR is shown by avoidance strategies, and such measures are not always possible [51]. Consequently, several studies were conducted and are currently ongoing to identify protective factors that can reduce the intensity or the frequency of the symptoms and/or prevent the onset of AR and AD.

Since oxidative stress is one of the risk factors involved in the atopic march, antioxidant molecules such as vitamin C, vitamin E, and to a much more debated extent, vitamin D, are quite promising. In the atopic-march-related context, such vitamins can indeed protect against oxidative-stress-induced cellular/tissue damage. For example, preclinical and clinical evidence has shown the protective role of vitamin E and vitamin D against asthma risk [52]. On the other side, an interesting study suggested that a maternal diet based on vitamin-C-rich fresh foods was associated with a reduced risk of infant wheezing in the first year of life [53].

Along with the importance of the immune system and microbiota composition, several pieces of evidence support the use of probiotics during the prenatal and postnatal periods as they may be beneficial to preventing and treating AD during childhood [54,55].

##### Symptomatic Treatments

Medicine-based therapies aiming at reducing the symptoms of allergies are often prescribed. They usually encompass corticosteroids and oral/topical antihistamines [56].

Since BE Barton et al.’s first delineation of mometasone furoate as a potent anti-inflammatory agent in 1991, corticoids have been usually employed to treat AR symptoms [57]. In this breakthrough in vitro study, the authors found that WEHI-265.1 murine myelomonocytic leukemia cells, when stimulated with lipopolysaccharide (LPS) and treated with this steroid, reduced their secretion of the two pro-inflammatory factors, IL-6 and TNF-α. Moreover, this drug also impaired IL-1 synthesis in LPS-stimulated murine peritoneal macrophages. Since then, inhaled glucocorticoids, the first line of treatment for AA [58], have also led to significant improvements in symptom management for AD. However, glucocorticoids are still associated with many side effects, and the clinical manifestations of allergies often re-appear after treatment withdrawal.

In addition to its role in immune and inflammatory cells directly related to allergy mediation, histamine also displays a wide range of biological functions mediated by its seven trans-membrane domain H1 receptors. Strategies for H1 receptor inhibition, thus, impact its classical functional effects, such as sensorineural stimulation, vascular dilatation, vascular permeability, and smooth muscle contraction in nasal/lower airways and gastrointestinal axis, as well as within the skin [59]. From a clinical standpoint, histamine H1 receptor blockers are, thus, commonly used to treat all types of itching and reduce pruritus [60]. However, their sedative effect is hard to manage for patients, as they affect decision-making, verbal learning, and psychomotor skills [61]. To palliate these inconveniences, non-brain-penetrating antihistamines such as fexofenadine and bilastine were developed and confirmed to not create sedative effects [56]. Consequently, they were even suggested to preferentially be chosen for the first-line therapy of mild AR. However, in the context of AD, no evidence has clearly shown that nonsedating antihistamines reduce itching or that sedating antihistamines provide any benefit in controlling the symptoms of AD, apart from better sleep management and the appearance of AD comorbidities, such as AR [62]. In any case, a study reported that the duration of the use of concomitant topical moderate-to-potent corticosteroids was reduced in AD infants treated with Cetirizine compared with a placebo, and that this cortico-sparing effect was statistically significant in children with a SCORing Atopic Dermatitis (SCORAD) score ≥25 [63].

##### Allergen-Specific Immunotherapies

Apart from symptom management treatments, desensitization therapies, also called “allergen-specific immunotherapy” (ASIT), have emerged as interesting options for treating diseases associated with the atopic march. Overall, the goal of these desensitization strategies is to directly act on the immune system to induce long-term immunological and clinical tolerance. The main cellular effects of these therapies reside in stimulating the secretion of anti-inflammatory cytokines from Treg and promoting the balance between the Th1 and Th2 cells while limiting IgE-mediated reactions. It is important to delineate that amongst the fourteen clinicians’ recommendations addressed in the Clinical Practice Guideline of the AR, immunotherapy implementation is encouraged for patients with AR who have not experienced adequate response to symptoms with pharmacologic therapy [22].

Many double-blind placebo-controlled trials and meta-analyses compiling ASIT’s results in the context of HDM-desensitization have reported the beneficial effects of this treatment modality for AD patients [64]. However, the lack of data reporting immunologic changes and serologic effects before/after treatment is still a research gap that needs to be filled to prove that ASIT rightly induces immune tolerance to allergens. Indeed, while a decrease in serum IgE is the main parameter expected to change, significant diminution in Der p-specific IgG4, IL-16, and thymus- and activation-regulated chemokine (TARC) were also reported in several ASIT studies [64]. More studies are needed in this field as treatment schedules, effective dosing ranges of allergens, safety profiles, and convenience for patients regarding administration routes for these therapies (including subcutaneous, sublingual, intra-lymphatic, epicutaneous, and local nasal) vary [65].

Overall, none of the above-mentioned options for treating atopic march-related diseases are ideal, as each has its limitations, is often cost-prohibitive for most patients, and none are completely effective, and/or devoid of unwanted side effects.

##### Cytokine Targeting as a Therapeutical Option

For similar reasons to ASIT, using cytokine preparations to balance the immune responses related to allergic reactions is another promising strategy. In particular, Th2-related cytokines such as IL-4, IL-5, and IL-13, which are well known to be responsible for the humoral immune response, were all investigated as targets of choice in developing targeted strategies for allergic desensitization.

In the context of airway hyperresponsiveness (AHR), the inhibition of such cytokines was already proven to be effective in controlling allergic symptoms. For instance, the anti-IL-4 receptor (IL-4R) α monoclonal antibody Dupilumab, which has the potency to impair both IL-4 and IL-13 signaling, has demonstrated good results as add-on therapy in patients with uncontrolled persistent asthma on medium-to-high-dose inhaled corticosteroids, in addition to a long-acting β_2_ agonist [66]. This antibody presented a favorable safety profile, increased lung function, and diminished severe asthma intensifications. Nevertheless, multiple other strategies aiming at blocking IL-4 or IL-4R activity and/or expression were developed to treat AA, as Maes et al. thoroughly addressed in their complete review on this subject [67].

The efficacy of Mepolizumab, a humanized monoclonal antibody against IL-5, was demonstrated in patients with uncontrolled severe eosinophilic asthma [68]. The rate of exacerbation was reduced by 47% among patients receiving a 75-mg intravenous dose and by 53% among those receiving a 100-mg subcutaneous dose every 4 weeks for 32 weeks. Moreover, in both treatment routes and doses groups, improvements in asthma control were noticed, as attested by forced expiratory volume in one-second (FEV_1_) values, St. George’s respiratory questionnaire (SGRQ) scores, and five-item asthma control questionnaire (ACQ-5) scores. In addition to being used as an add-on therapy in adults with severe uncontrolled asthma with an eosinophilic phenotype, this anti-IL-5 was also employed for patients with severe corticosteroid-dependent asthma [69]. A multidisciplinary task force of asthma experts’ recommendations even recently suggested using a blood eosinophil count cut-off point of ≥150 cells/µL as an anti-IL-5 initiation guide in adults with severe asthma and a history of prior asthma exacerbations. It was shown that in patients requiring daily oral glucocorticoids to maintain asthma control, Mepolizumab had a significant glucocorticoid-sparing effect, as it led to a likelihood of a reduction in the glucocorticoid–dose stratum 2.39 times greater than compared with a placebo [70]. Moreover, low-dose Mepolizumab (100 mg q4w) showed effects in reducing the eosinophil blood counts and in improving both the sinonasal scores and the asthma symptoms of patients with severe asthma and active eosinophilic granulomatosis with polyangiitis (EGPA) [71].

Apart from IL-4 and IL-5 targeting, the blockade of IL-13 and IL-6 receptors was also assessed, but these options require more research. Assays on the anti-IL-13 antibody Tralokinumab reported a possible positive effect in a specific sub-group of patients with severe uncontrolled asthma and levels of serum dipeptidyl peptidase-4 (DPP-4) or periostin higher than the baseline median population [72]. In addition, over a 52-week trial, the effects of Lebrikizumab, another anti-IL-13 antibody, were also demonstrated, as it reduced the exacerbation rates in biomarker-high patients (periostin ≥50 ng/mL or blood eosinophils ≥300 cells/µL), even if the results were not significant [73]. A small study conducted in Switzerland assessed the effect of Tocilizumab, a monoclonal antibody against the IL-6 receptor, in three patients affected with severe excoriated AD of the extremities and the trunk [74]. Despite the small number of patients and the open design without a control group or period, Tocilizumab treatment significantly improved the AD clinical activity, as assessed by the eczema area and severity index (EASI), which encompasses erythema, induration, excoriation, and lichenification of the skin, but was associated with bacterial superinfection.

Moreover, as more knowledge is now available about the implication of IL-17, IL-19, IL-33, and TSLP in AD pathogenesis, directed therapies against these cytokines and factors were also developed and emerge as interesting alternatives for patients, even if more research still needs to be completed in this area [75].

Pursuing a multi-target approach, micro-immunotherapy (MI) is another immune-targeted strategy that can be employed for allergic patients. Indeed, as many pleiotropic cytokines and immune factors are involved in the initiation and the perpetuation of the full panoply of immune responses leading to allergic inflammation, adopting a multi-target therapy to act on several facets of the allergic mediation at a time constitutes a promising strategy. Therefore, instead of inhibiting or blocking a specific cytokine and/or its signalization, MI employs several immunoregulators, mainly in the form of recombinant cytokines, at low doses (LD) and ultra-low doses (ULD) to minimize the secondary effects, acting simultaneously on multiple targets, thus, modulating their related pathways.

In the Section 2 of this manuscript, we will first introduce the MI medicine 2LALERG^®^ and provide more detail on its composition and main characteristics. We will then discuss its potential beneficial use in the context of the atopic march.

## 2. Micro-Immunotherapy and 2LALERG^®^: The Use of Immune Factors at Low Doses and Ultra-Low Doses

### 2.1. Presentation of Micro-Immunotherapy and 2LALERG^®^ Formulation

The MI medicine 2LALERG^®^ is considered a homeopathic medicinal product composed of LD and ULD of immune modulators (mostly cytokines) impregnated on sucrose-lactose pillules (also called globules) for oromucosal administration. The composition of the medicine, expressed in centesimal Hahnemannian dilutions (CH), also includes a specific nucleic acid (SNA^®^), SNA^®^-HLA II, which consists of nucleic acid sequences especially designed to target HLA-II. The globules, packed into capsules intended to be opened, are to be poured under the tongue and taken in the morning in a fasted state. Oromucosal administration is intended to reach the numerous immune cells in the oral–pharyngeal mucosal and gastrointestinal tracts [76,77], which communicate in a continuous cross-talk with the systemic immune system [78]. As with all MI medicines, one of the specificities of 2LALERG^®^ resides in the sequentiality of its delivery. A complete sequence of 2LALERG^®^ encompasses ten different capsules, each with a unique composition, and is intended to be taken following a numerical order, with capsule 1 taken on day 1, capsule 2 on day 2, etc.

The specific combination of immune regulators at LD and ULD found in 2LALERG^®^ is intended to gently modulate their expression and related pathways within the body. Indeed, the LD of bioactive molecules (ranging from 3 to 5 CH) are intended to mimic the low physiological doses found in the organism and are employed to induce their biological effects, thus, acting as agonists [79,80]. For instance, recently published results reported the immunostimulatory and co-stimulatory effects of the unitary medicine γ interferon (IFN-γ) (4 CH) on different cell subpopulations [79]. On the other hand, the ULD (from 6 CH up to 30 CH) used in MI are employed to display modulatory responses (from 6 CH) and/or down-regulatory responses (from 12 CH and beyond) towards the expression of the concerned targets. These assumptions are supported by the fact that IL-1β (27 CH) and TNF-α (27 CH) both exhibit anti-inflammatory effects, as each could reduce the secretion of IL-1β and TNF-α in a model of LPS-stimulated human primary monocytes and the monocyte-like THP-1 cell line [81]. Moreover, a MI formulation that includes ULD of IL-1β and TNF-α as a part of its active ingredients also displayed anti-inflammatory properties in a model of LPS-stimulated human primary monocytes and in vivo, in a model of rheumatoid arthritis [82,83].

The overall formulation of 2LALERG^®^, also provided in Table 1, is as follows: IL-1β (17 CH), IL-4 (17 or 27 CH), IL-5 (17 CH), IL-6 (17 CH), IL-10 (17 CH), IL-12 (9 CH), IL-13 (17 CH), TNF-α (17 CH), tumor growth factor β (TGF-β) (5 CH), *pulmo-histaminum* (15 CH), and SNA^®^-HLA II (18 CH).

In summary, all the above-mentioned ingredients present in the formulation of 2LALERG^®^ at the 9 CH to 27 CH ranges are supposed to modulate/down-regulate specific targets. On the opposite side of the spectrum, the unique active ingredient employed at 5 CH is intended to act as an agonist. The following parts of this review aim to discuss, through an updated body of the literature, the rationale and the therapeutic potential behind the use of these cytokines and immune factors at low and ultra-low amounts to counteract the symptoms of atopic conditions such as AD and AR. In addition, the preclinical and clinical results of the studies already conducted on 2LALERG^®^ in the context of AR will also be described.

### 2.2. Rationale behind the Cytokine Combination

#### 2.2.1. Cytokine Implication at Several Steps of the Atopic-March-Related Immune Response

##### Interleukin-1β and Neutrophils

Interleukin-1β is one of the cytokines employed within the active ingredients of 2LALERG^®^. It is used at ULD (17 CH) (see Table 1) to down-regulate its expression and/or function, as well as the activation of its related pathways.

This interleukin was reported to be one of the first inflammatory factors involved in allergic responses, as its level was significantly increased in nasal secretions of allergic subjects after antigen provocation compared with pre-challenge values in the context of immediate and late-phase allergic reactions [84]. Interestingly, a correlation was also found between IL-1β levels within the nasal secretion and the corresponding total symptom scores during these reactions. In their study conducted in Ulsan of Korea, Han et al. identified IL-1β as a strong risk factor for developing moderate to severe, persistent AR, thus, highlighting that it could be employed as a biomarker for the exacerbation or activation of allergic diseases [85].

Several transgenic murine models were also employed to document the implication of IL-1β in allergic responses. In particular, a study showed that AHR was dramatically reduced in IL-1α/β^−/−^ BALB/cA background mice subjected to repeated OVA/phosphate-buffered saline (PBS) sensitization without alum, in comparison to wild-type animals [86]. Accordingly, this study delineated the importance of IL-1β in the activation of Th2 cells, as its authors also demonstrated that antigen-specific T-cell proliferative responses, T-cell secretion of IL-4 and IL-5, as well as B cell secretion of antigen-specific IgG1 and IgE were significantly reduced during the course of AHR in IL-1α/β^−/−^ mice compared with wild-type mice. By studying pulmonary immune responses in IL-1R1^−/−^ animals, Schmitz et al. found that IL-1 elicited asthmatic responses in a mild model of allergic asthma induced by repeated sensitizations with intraperitoneal injections of low doses (10 µg) of OVA, without any adjuvant [87]. Their results showed that, in these animals, pulmonary and serum antibody responses, including IgM, IgG, IgA, and IgE isotypes, were considerably reduced compared to control mice. In addition, Th2-type inflammation, CD4^+^ recruitment, eosinophilia, and goblet cell mucus production decreased in this murine genetic background compared to wild-type animals. The role of IL-1 in the development of AR was also assessed in a toluene-2,4-diisocyanate (TDI)-induced guinea pig model, in which the blockade of the IL-1 pathway was achieved through intra-muscular injections of the IL-1 antagonist receptor (IL-1Ra) [88]. The results showed improvements within the manifestations of the AR symptoms in the treated group compared with the control group, as less edema and decreased histamine levels were found in the nasal mucosa, concomitantly with reduced IgE levels in the blood.

In addition to the aforementioned wide range of cellular and systemic effects of IL-1β in the context of allergy, we wanted to focus our attention on the role of this cytokine in the neutrophil subpopulation. While neutrophils have long been considered unsophisticated, terminally differentiated cells only implicated in acute bacterial infections, emerging evidence suggests that some neutrophil subsets might influence the characteristic course of AR inflammation [89]. Indeed, when activated with LPS, TNF-α, and IL-8, these cells were able to help in T-cell activation in a cell–cell contact-dependent fashion and up-regulate eosinophilic migration. Moreover, in patients with intermittent AR, a single allergen provocation induced an early-phase response dominated by neutrophils [90]. These authors also reported that both the total nasal symptom score and the secretion score were correlated with the number of neutrophils in lavage fluids at 1 h post-allergen challenge, suggesting that these cells may even have a pivotal role in the promotion of nasal secretion in AR patients.

In addition, neutrophils are the first immune cells to infiltrate AD skin and are implicated in the early itch behaviors related to AD [91], suggesting that therapeutic targeting of this cellular population could be beneficial in the context of atopic march management. For this reason, modulation of the IL-1β pathway was tested and provided interesting results regarding its effect on neutrophils. Using an LPS-induced airway neutrophilia model of acute neutrophilic asthma exacerbation, a double-blinded, placebo-controlled, crossover study evaluated the effects of anakinra, a recombinant form of human IL-1 receptor antagonist (IL-1 Ra), in suppressing airway inflammatory responses [92]. The results showed that the IL-1β signaling blockade significantly decreased the percentage of neutrophils/mg in sputum compared with the placebo.

**Figure 3:**
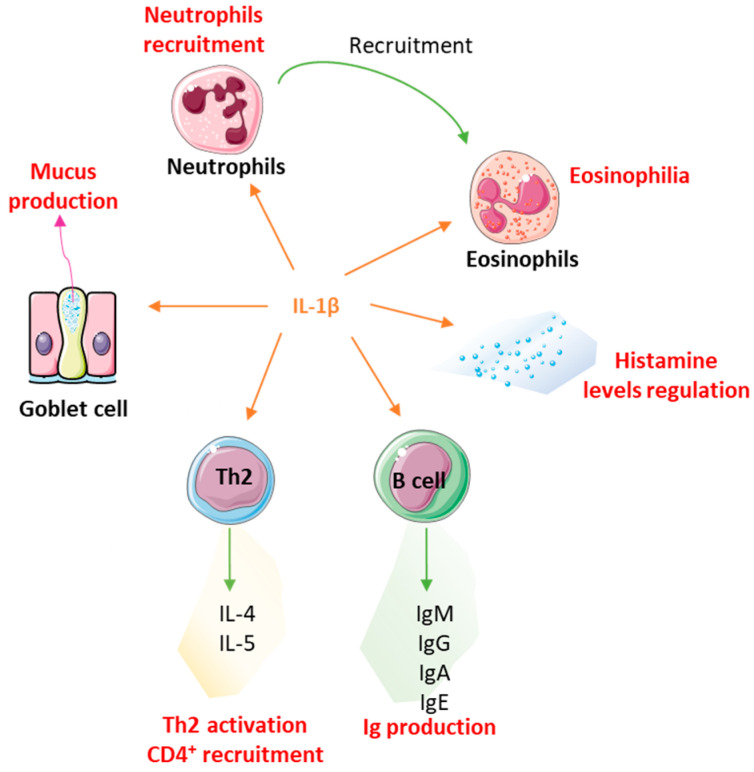
The pro-inflammatory cytokine IL-1β is implicated in regulating atopic-march-related immune responses. This illustration provides a non-exhaustive representation of the atopic-march-related processes in which IL-1β is involved, as covered in previous paragraphs. Ig: immunoglobulin.

Overall, the strong involvement of IL-1β in allergic diseases (Figure 3) means that treatments aimed at down-regulating this cytokine and its signaling pathways are attractive therapeutic options during acute and chronic manifestations to reduce the symptoms of IgE-mediated inflammation. Furthermore, it is also worth mentioning that, in addition to the context of an allergy, MI has previously shown IL-1β-related anti-inflammatory potential and the capability to down-regulate IL1-β expression when using this same cytokine in ULD [81,82,83].

Nevertheless, numerous other ILs and immune factors also play a role in the complex etiopathogenesis of the atopic march, and a multi-target therapeutic strategy that has several regulatory effects on those factors, such as that employed in MI, could be of great interest and beneficial for patients.

##### The Multistep Process of the Atopic March Is Mediated by IL-4, IL-5, IL-6, and IL-13

Furthermore, 2LALERG^®^ includes ULD of IL-4, IL-5, IL-6, and IL-13 (Table 1) to down-regulate the expression and/or secretion of these well-known allergy-related ILs. The following paragraphs provide an overview of these cytokines’ main roles in the physiology of atopic-march-related conditions. In particular, their mutual interactions, their implication in skin barrier integrity, and eosinophils functions are developed.

- IL-4, IL-5, and IL-13: Implications in Skin Barrier Functions

IL-4 and IL-13 are very similar cytokines in that they are encoded by adjacent genes located on human chromosome 5q, share common regulatory elements (GATA-3), and also, transmit a signal through a similar receptor complex (IL-4Rα/IL-13Rα1) [93]. Despite these similarities, IL-4 and IL-13 each have a role in AA responses, particularly regarding the temporal aspect of immune response mediation. Indeed, IL-4 appears to be more involved in initiating the allergic airway reaction, as it regulates Th2 cell proliferation/survival and IgE synthesis and mediates humoral responses. On the other hand, IL-13 is implicated in the late effector phase of the allergic manifestations, leading to airway remodeling characterized by AHR, airway occlusion due to mucus hypersecretion, plugging and deposition of Charcot–Leyden crystals by activated eosinophils, goblet cell hyperplasia, airway smooth muscle alterations, epithelial hypertrophy, and sub-epithelial fibrosis-related diminished lung functions [94]. Regarding these epithelial-related dysregulations, a glimpse into the relationship between IL-4, IL-5, IL-13, and the epithelial aspect of the atopic march was needed, and several pieces of evidence suggest that IL-4 and IL-13 could act in concert with the epithelial-cell-derived cytokine TSLP, an IL-7-like cytokine, previously reported to potently stimulate the production of Th2 cytokines by human mast cells, in synergy with IL-1 and TNF-α [95]. TSLP is associated with AR in children with AA [96] and is a key cytokine involved in the skin inflammation related to AD, and its role as a Th2 activator has also helped better characterize the immune cascade orchestration of these responses.

Concerning the IL-4/IL-5/IL-13 axis, it was shown that TSLP-promoted IL-4 induction in CD4^+^ cells in skin-draining lymph nodes was driven by a fine-tuned sequential cooperation between dendritic cells (DC), T-cells, and basophils through a “DC-T cells-basophils-T cells” cascade [97]. In addition, studies reported that DC treatment with TSLP induced the production of IL-4, IL-5, and IL-13 from naïve CD4^+^ T-cells upon co-culture [98].

Moreover, IL-4 and IL-13 may play a role in regulating the skin-barrier-related gene filaggrin, as its expression was significantly reduced in acute AD skin and in keratinocytes differentiated in the presence of these cytokines [99]. As mentioned in the introductory part of this review, the skin barrier defects found in AD patients are permissive to further bacterial infections and their inherent complications. Species such as *Staphylococcus aureus* are commonly found as AD patients’ skin colonizers and are reported to be involved in the development and aggravation of AD symptoms [100]. Normal keratinocytes constitutively express antimicrobial peptides, which can kill *S. aureus*, such as human-β defensin-3 (HBD-3). It was reported that AD keratinocytes expressing similar HBD-3 levels to their unaffected counterparts could not mobilize HDB-3 efficiently to kill *S. aureus* [101]. Interestingly, this study also reported that antagonizing the Th2 cytokines IL-4, IL-10, and IL-13 led to a restoration of HBD-3 mobilization from the keratinocytes’ cytoplasm onto the bacterial surface, which was three-fold more important than control conditions.

Such results, thus suggest that a therapeutic strategy aiming at reducing IL-4, IL-10, and IL-13 could also, beyond the direct immune-cell-related role of these cytokines, improve the skin barrier integrity of patients with AD.

- IL-5, IL-13, and Eosinophils

Cytokines, especially IL-5 and IL-13, were shown to affect eosinophils, another immune cell subpopulation highly involved in the atopic march process. Pope. et al. reported that a 10-day, daily intranasal administration of IL-13 in BALB/c mice led to preferential eosinophil recruitment into the bronchoalveolar lavages fluids (BALF) compared with a saline-administered control group [102]. This effect depended on IL-5, as the IL-13-induced lung eosinophilia was markedly impaired in IL-5-deficient mice. The chemo-attractive capabilities of IL-13 were also highlighted in another study, in which intratracheal instillation of 500 ng of recombinant murine IL-13 into F344 Fisher rats led to airway neutrophil recruitment and induced TNF-α expression in the airway epithelium-infiltrating-neutrophils [103]. These authors attributed this local effect to the fact that IL-13 induces an airway epithelial IL-8-like chemoattractant. Interestingly enough, in the model from Pope. et al., IL-13 did not affect the circulating levels of eosinophils and only mediated a localized effect within the lungs. Conversely, while IL-13 was also reported to induce mucus secretion, this mechanism was not found to be IL-5-dependent [102]. Using a guinea pig model and light microscopy techniques, Palframan et al. reported that IL-5 stimulates the rapid trafficking of eosinophils from the bone marrow hematopoietic compartment to the sinuses in a dose-dependent manner, thus, highlighting the implication of this cytokine at the early stages of the allergic inflammatory response [104]. Interestingly, eosinophils mobilized by IL-5 display a shed expression of L-selectin and an increased expression of the β_2_ integrin. Finally, in their model of low-density bone marrow cells stimulated with 10 ng/mL IL-5, Fulkerson et al. reported that brief exposure to this cytokine was sufficient to induce a cooperative cytokine network, promoting terminal eosinophil differentiation in the absence of further IL-5 stimulation [105]. Furthermore, these authors also demonstrated that IL-4, IL-6, IL-9, IL-10, IL-12p40, and TNF-α were detected in the supernatant of these stimulated cells, suggesting that these cytokines may contribute to the final stages of eosinophil differentiation.

Together with the data available concerning the implication of IL-4, IL-10, and IL-13 in skin barrier integrity, these results provide more information concerning the role played by IL-5 and IL-13 in the mediation of eosinophil recruitment and activation.

##### Histamine Secretion: A Process Orchestrated by Cytokines

- Mast Cells, Basophils, and Histamine: An Introduction

Histamine is an allergic mediator involved in the late phases-related manifestations of an allergy and in acute allergic reactions. Alongside heparin, tryptase, and the lysosomal-associated membrane protein CD63, this histidine derivative is one of the typical degranulation markers secreted by immune cells such as mast cells and basophils [106]. As both originate from pluripotent hematopoietic stem cells, mast cells and basophils share many features. This includes their common expression of FcεR1, their capacity to express membrane markers such as lipid metabolites leukotriene C4 (LTC4) and prostaglandin D2 (PGD2) after activation, as well as their ability to secrete Th2 cytokines and histamine [106,107]. Despite these similarities, mast cells and basophils are still two very distinct cell types, which differ in their maturation sites, primary location, lifespan, granules phenotype, or lipid mediators’ production, for instance. In addition, it is also thought that these cells operate at different stages during the course of the allergic reaction. Indeed, while tissue-resident mast cells appear to be essential for mediating immediate hypersensitivity, the direct homing of basophils to the allergic site is, for its part, one of the characteristics of late-phase allergic inflammation.

The implication of cytokines in orchestrating the activation of these two cells’ subtypes and their fine-tuned control of histamine release has been widely studied and documented. The following sub-sections of this review, thus, discuss their roles in this particular context and the rationale behind the multi-target approach of 2LALERG^®^ aimed at regulating their expression and related pathways.

- IL-1β, IL-4, IL-5, IL-13, and TNF-α: Implications in the Stimulation of Mast Cells and Basophils

Several lines of evidence support the role of the ILs used at ULD in the 2LALERG^®^ in mediating histamine release. Given their 1988 publication, Haak-Frendscho et al. can be considered pioneers in determining the nature of the histamine-releasing factors secreted by human lymphocytes and alveolar macrophages [108]. In their work, these authors assessed the responsiveness of basophils subjected to human recombinant IL-1, or TNF, through histamine release measurement. Despite the heterogeneity found between the eleven donors, this study reported that basophils from approximately 30% of the subjects responded to either IL-1 or TNF by releasing histamine, and noticed that the strongest responses were elicited by IL-1. In line with these results, the stimulatory effect of TNF-α on histamine secretion was confirmed in a model of cutaneous mast cells, in which it occurred in a time- and concentration-dependent manner [109].

This first piece of data concerning IL-1 was then uncovered by Bischoff et al., who also included IL-5 in their studies [110]. They found that when basophils were pre-incubated with either 100 ng/mL IL-1β or with 10 ng/mL IL-5 before stimulation with complement cleavage products C3a, C5a, anti-IgE, or human recombinant neutrophil-activating peptide 1 (r-NAP-1), they displayed an enhanced histamine release compared with their non-pre-incubated counterparts. Interestingly, this study also stipulated that IL-5-priming allowed basophils to generate leukotriene LTC4 when further stimulated with C3a or with anti-IgE, a factor that was barely produced without IL-5. Such results resonate with the clinical data, as IL-5 was detected in the nasal secretion of allergic patients during both the immediate and the late-phase reactions after an allergen challenge [84]. With this in mind, it is interesting to note that anti-IL-5 therapy effectively reduces the number of epithelial mast cells in patients with eosinophilic esophagitis [111].

In parallel, the role of IL-4 and IL-13 in the mast cell- and basophil-related context was widely documented by employing numerous human and mice models of allergy. For instance, by using their IL-13 transgenic mice model, Fallon et al. found that, once sensitized, the serum of these mice elicited substantial basophil degranulation in the presence of egg antigens, in comparison to those from naive or sensitized wild-type mice [112]. As diverse stimuli, including IgE, activate basophils [113], these data are corroborated by the fact that in T and B cells isolated from AA patients, IgE production is more dependent on endogenous IL-13 than in normal controls [114]. Additionally, once expanded in vitro, these patients’ cells display enhanced production of IL-13 compared to controls (385 pg/mL vs. 175 pg/mL). IL-4 is also a key factor involved in regulating human mast cells. For instance, the association of IL-4 and stem cell factor (SCF) was found to cause a profound enhancement in the proliferative capabilities of intestinal tissue-isolated mast cells [115]. Finally, in this model, the release of histamine in response to IgE-receptor crosslinking was more pronounced when 10 ng/mL IL-4 was added to the culture medium.

The overall evidence discussed suggests that a multi-target cytokine strategy aiming at reducing IL-1β, IL-4, IL-5, IL-13, and TNF-α could, in turn, diminish the release of histamine. Since this factor is one of the main allergic mediators causing the typical clinical manifestations of allergies, this therapeutic strategy could ultimately offer better efficacy than a single-target therapy in managing atopic-march-related diseases.

- What About the Role of IL-6?

While the role of IL-6 in the atopic march remains controversial and is not as well documented as the above-mentioned factors, this pleiotropic cytokine is involved in inflammation regulation at both humoral and cellular levels [116,117]. The implication of this cytokine in atopic reactions was pinpointed by the fact that peripheral blood T-cells isolated from AD patients secrete significantly higher levels of IL-6 than their normal donor counterparts [118]. Additional pieces of evidence were provided by Gosset et al., who showed that the presence of IL-6 in nasal secretion was observed as both an immediate (15 to 90 min post-allergen challenge) and a late-phase reaction (90 min to 9 h 30 min post-allergen challenge) [119]. Moreover, some evidence suggests that targeting this cytokine could impede mast cell/basophil-related histamine release.

It was reported that peripheral-blood-derived cultured mast cells incubated with both IL-6 and SCF for one week displayed a higher propensity to IgE-dependent histamine release and a higher intra-cellular histamine content than SCF-only incubated control cells [120]. Moreover, it was also noted that anti-IgE-dependent histamine secretion was elevated more when mast cells were cultivated in the presence of IL-6, SCF, and IL-4 together, compared with the combination of only SCF + IL-6.

In terms of therapeutic considerations, it is also worth mentioning IL-6′s suggested role in anti-histamine resistance, which was uncovered in the context of acute urticaria [121]. In this study, 9 out of 16 patients showed elevated IL-6 and C-reactive protein (CRP) plasma levels, and all were resistant to classical anti-histamine treatment, leading them to receive steroid administration to cure the skin wheals. Conversely, in the patient group displaying normal IL-6 blood ranges, most positively responded to the H1 receptor antagonist, Cetirizine. In accordance with these results, Desai et al. reported that IL-6 enhanced the proliferation and the maturation of human mast cells, as indicated by an increase in cell size, granularity, and chymase protease content [122]. Finally, the same authors also showed that IL-6 led to an increase in FcεR1 ligation-induced degranulation, as well as higher production of granulocyte-macrophage colony-stimulating factor (GM-CSF) and IL-8.

- Inhibiting IL-10 to Reduce Histamine-Releasing Processes

Interleukin 10 is a pleiotropic cytokine known to display either anti- or pro-inflammatory capabilities during immune responses. Some studies have reported its role in promoting eosinophilia, AHR, mucus metaplasia, and IL-5 production during allergic responses. IL-10 was overexpressed in large mononuclear cells in the dermal infiltrate of AD lesions, and its spontaneous release from peripheral blood mononuclear cell (PBMC)-derived adherent cells was also higher in AD donors than in healthy controls [123]. Moreover, an in vivo study suggested the implication of this cytokine in eosinophil recruitment. The authors used an IL-10^−/−^ murine model of AD established by epicutaneous sensitization with OVA on tape-stripped-skin and reported a decrease in eosinophil infiltration, a reduced expression of eotaxin (an eosinophil chemoattractant), IL-4, and IL-5 in the OVA-sensitized skin sites of these animals [124].

Interestingly, some evidence has shown that IL-10 may play a role in histamine-releasing mechanisms. Polukort et al., in their food-allergic IL-10^−/−^ murine model, showed that the intestinal anaphylaxis of their transgenic model was significantly weaker than in the wild-type BALB/c OVA-challenged mice in terms of mast cell activation and Th2 cytokine production [125]. Jejunal tissue analysis showed that very few to no degranulated mast cells were present in the OVA-challenged IL-10^−/−^ mice compared to the control group, even though similar levels of IgE were observed in both groups.

- Histamine as a Self-Regulator of its Own Release

*Pulmo-histaminum* (15 CH), employed in the formulation of 2LALERG^®^, could lower the levels of histamine secreted: this assumption has been sustained by a multi-site study that demonstrated the reproducibility of human basophil inhibition when treated with high dilutions of histamine [126]. Such dilutions, ranging from 10^−30^–10^−38^ M, consistently modulated basophil activity, as reported by Alcian blue staining and confirmed by flow cytometry analysis methods. From a clinical standpoint, data are scarcely available about *histaminum* when used at ULD as a single ingredient, and more research is needed. However, the efficacy of a combination of cat saliva 9 CH and *histaminum* 9 CH on cat allergy was assessed in a double-blind, randomized, placebo-controlled study conducted on cat-allergic adults with positive results on cat allergy skin-prick tests [127]. The prick test measures reported a significant reduction in the diameter of the wheal in the group that completed the 4-week treatment with the ULD preparation of cat saliva and *histaminum* compared with the placebo-treated one. Based on this trial, and since pet allergies are also considered a major risk factor for the development of AR and AA [128], lowering histamine levels through the use of *pulmo-histaminum* (15 CH), as employed in the formulation of 2LALERG^®^, might be beneficial to managing acute symptoms related to atopic march manifestations.

##### The Case of TGF-β: Why Targeting This Factor Could Contribute to the Management of Allergic Diseases

The formulation of 2LALERG^®^ relies on the presence of TGF-β at the stimulatory dose of 5 CH to exert a gentle boosting of TGF-β related pathways during allergic reactions.

An analysis of biopsy specimens from pruritic plaque skin lesions taken from patients with chronic dermatitis revealed a decreased expression of both TGF-β1 and TGF-β2 isoforms compared with healthy individuals’ skin samples [129]. These data can be correlated with the study from Arkwright et al., in which they highlighted the association between AD and a low-producer *TGF-β1* genotype and suggested that the relative deficiency of TGF-β1 in the skin could result in the immune response characteristic of AD [130].

Mechanistically, TGF-β undergoes a proteolytic process to activate, as its binding to latency-associated protein (LAP) or latent-TGF-β-protein (LTBP) keeps it inactive [131]. Once activated, TGF-β can bind its heterodimeric trans-membrane receptor complex, composed of type I and type II subunits, and then trigger the intra-cellular canonical Smad signalization [132], in which, in vertebrates, five receptor-associated Smads (R-Smads1, 2, 3, 5, and 8), one common Smad (Co-Smad4), and two inhibitory Smads (I-Smads6 and 7) are involved. Smad-independent signalization networks were also reported and include several offshoots of mitogen-activated protein kinases (MAPK), such as p38 MAPK, c-Jun N-terminal kinase (JNK), Rho-like GTPase, phosphatidylinositol 3-kinase/Ak strain transforming (PI3K/AKT), and extra-cellular signal-regulated kinase 1/2 (ERK 1/2) [133].

From a physiologic standpoint, TGF-β displays its anti-inflammatory and immunosuppressive properties at several levels of immunity, either acting on components of the innate responses such as natural killer (NK) cells [134,135], or by regulating the adaptive side of immunity, as reflected by its ability to inhibit immune cell differentiation (Th1, Th2, and B cells) and cytokine production. Multiple mechanisms of action explaining its effect on cell differentiation were studied, and numerous pieces of evidence have shown that TGF-β is a potent inhibitor of the two transcription factors T-bet and Gata-3, which are necessary for the differentiation process of Th1 and Th2 cells, respectively [136].

On the other hand, TGF-β is critical for the development and differentiation of Treg [137]. The effect of TGF-β on immune tolerance was studied by Lin et al., who assessed the role of Treg cells in the context of AR [138]. Their study showed that the adoptive transfer of AR CD8^+^ Treg increased the levels of TGF-β in mice nasal lavage fluids, which was associated with a reduced number of sneezes and nasal rubbing incidents in the treated animals. Interestingly, Singh et al. documented the critical implication of B cells in the resolution of allergic airway diseases, thanks to their B cell deficient murine model adoptively transferred with B cells recovered from hilar lymph nodes of OVA-sensitized-mice that developed a local inhalational tolerance through chronic continuous antigen exposure [139]. These authors found that the transferred B cells inhibited the in vivo bronchoalveolar lavage (BAL) leukocytosis and eosinophilia associated with allergic airway diseases and that the regulatory effects exerted by these so-called B regulatory (B reg) cells were mediated by TGF-β, as it induced conversion of CD4^+^CD25^−^ T effector cells into functionally suppressive CD4^+^CD25^+^Foxp3^+^ Treg cells.

Since TGF-β signaling pathways involve Smad3 proteins, the implication of this factor during allergic reactions was assessed in a Smad3^−/−^ murine model of AD in which dermatitis was induced by epicutaneous application of OVA, applied in a patch to tape-stripped skin [140]. Interestingly, in this study, an increased number of mast cells was found in the OVA-sensitized skin of Smad3^-/-^ mice compared with OVA-sensitized WT mice. In addition, OVA-specific IgE levels were also found to be significantly elevated in these animals compared with WT.

#### 2.2.2. Human Leukocytes’ Antigen Dysregulation in Atopic-March-Related Allergies

Cytokines are not the only immune factors that regulate cellular communication in allergic-related reactions, as numerous receptors play a role in these unwanted immune responses. Human leukocyte antigens (HLA), the major histocompatibility complex (MHC) class II molecules, are another factor.

Three different isotypes of MHC co-exist in humans—namely, HLA-DP, HLA-DQ, and HLA-DR—and their role is to help professional antigen-presenting cells (pAPC), such as dendritic cells, macrophages, or B cells, to present exogenous peptides to CD4^+^ T-cells, though T-cell receptor (TCR) interaction [141]. Such interactions are crucial to (i) initiate humoral immune responses and (ii) generate memory T-cells, which are two characteristics of adaptive immunity that should be minimized during allergic manifestations.

Several studies have reported that the immune cells implicated in atopic-march-related manifestations exhibited up-regulation in MHC-II expression. In particular, it was shown that the number of both HLA-DQ^+^ dendritic- and lymphocytic-morphology HLA-DR^+^ cells increased in the epithelium and the lamina propria of AR patients during allergen provocation [142]. Moreover, in AR, the HLA-DR^+^ and CD11c^+^ dendritic cells’ capability to penetrate beyond the well-developed epithelial tight junctions of the human nasal mucosa suggests that those cells could easily access the antigens, facilitating the triggering of the allergic reaction [143]. Apart from pAPC, keratinocytes were also shown to express HLA-DR in IFN-γ-inflamed-culture conditions [144], a mechanism that may contribute to the recruitment of immune cells in skin lesions during AD. Interestingly, as research constantly evolves, new subsets of immune cells are continuously discovered. For instance, initially identified as a subset of TNF-α and iNOS-expressing inflammatory dermal dendritic cells in psoriasis, the so-called 6-sulfo LacNAc-expressing monocytes (slanMo), which were also thought to play a role in the pathogenesis of AD, were shown to be HLA-DR^+^ when phenotyped into lesional AD skin samples [145].

The recently described subclass of innate lymphocytes ILC2, as mentioned in this paper’s introduction, display important functions in regulating immunity and homeostasis, specifically in the lungs [146], and are involved in the etiopathogenesis of airway allergic diseases. In terms of the role of this cell population and the implication of its HLA-DR expression in allergy and asthma, the results of a single-blind, placebo-controlled clinical study merit our attention. Investigators assessed the effects of the intranasal administration of the triamcinolone acetonide corticosteroid on this ILC2 population [147]. Interestingly, when assessed 24 h post-nasal-allergen challenge, such treatment significantly decreased IL-5^+^/IL-13^+^ ILC2s, as well as HLA-DR^+^ ILC2 within the nasal mucosa, thus, highlighting the fact that the attenuation of HLA-DR expression by ILC2 may be another mechanism of action of corticosteroids in modulating the adaptive immune responses in airway allergies that MI can exploit.

### 2.3. Preclinical and Clinical Pieces of Evidence on the Efficacy of 2LALERG^®^ in the Context of AR

In their murine model of allergic respiratory disease sensitized with birch pollen extract, Floris et al. showed that ten days of daily oral gavage with 2LALERG^®^ not only significantly reduced the total number of cells in the BALF, but also diminished the Th2 cytokine IL-13 level [148]. Moreover, some interesting trends were found: eosinophil, IL-4, and IL-5 levels were also reduced in BALF, and histological analysis in the lung revealed less inflammatory infiltration and lower mucus production in the treated group compared to the control group. Finally, the circulating levels of IL-5 and IgE were significantly reduced in the treated group, thus, showing an effect of the medicine at both local and systemic levels.

Interestingly, a double-blind, placebo-controlled study conducted in Belgium with 41 participants (from 6 to 41 years old) has proven the safety and provided preliminary clinical data concerning the efficacy of 2LALERG^®^ in the treatment of the AR during the grass pollen season’s peak [149]. Even though no significant improvement in the daily symptom score (assessed by the total 5 symptoms score (T5SS) questionnaire) was shown, a significant decrease in rescue medication consumption, as well as an improvement in the global symptom and medication score, were shown in the active group compared with the placebo one. In addition, the number of days free of any rescue medication in the active group was significantly decreased despite the small number of participants. Rescue medicines included oral/local antihistamines, local corticoids, and eyedrop cromoglycates, whose side effects can comprise convulsions, headaches, hepatic insufficiencies, dry mouth, asthenia, itching, corneal calcifications, and aerial upper tract infections including pharyngitis and nose/throat irritations. A second double-blind, placebo-controlled study performed afterward on 102 adult (≥18 years old) participants did not prove the superiority of the medicine compared to the placebo [150]. This might be due to the heterogeneity of the concomitant drugs taken during the study and the eventual comorbidities, which often occur in adult patients with AR [151,152] and may have added extra confounding factors to the clinical study.

Further clinical studies are recommended and should consider poly-medication and eventual comorbidities as crucial factors in the pathogenesis of atopic march diseases and the patient’s responsiveness to the treatment.

Preclinical research studies are also needed, as they can provide important additional information about the mode of action of 2LALERG^®^ that, in turn, might be useful in the set-up of future clinical studies. However, the hypothesized mode of action of MI medicines, as previously discussed in published articles, mainly involves: (i) the capacity of all organisms, cells, and tissues, particularly the immune system, to respond to a multitude of stimuli, in a wide range of concentrations, according to a non-linear dose–response curve, also called biphasic or hormetic response; (ii) the presence of active substances in LD-based MIM [79], as well as sub-micron particles, possibly containing starting materials, in ULD-based MIM [81].

## 3. Conclusions

**Figure 4:**
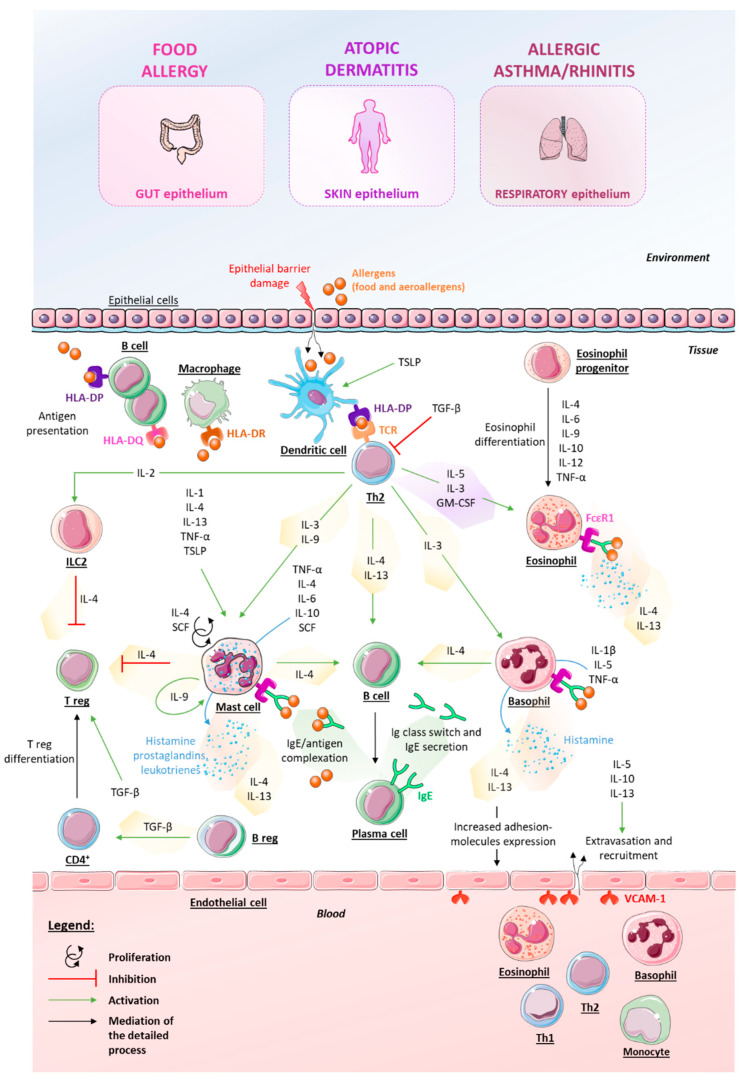
Recapitulative scheme of the immunological processes involved in atopic-march-related diseases. The top panel highlights that the illustrated reactions can occur in the gut, skin, or respiratory epitheliums, depending on the localization of the atopic symptom’s manifestations. This non-exhaustive representation aims to highlight the involvement of each cytokine and immune factor used in the formulation of the 2LALERG^®^ to show the rationale behind the use of LD/ULD of these components to act against allergic-related reactions. B reg: regulatory B cells; GM-CSF: granulocyte-macrophage colony-stimulating factor; HLA: human leukocyte antigen; Ig: immunoglobulin; IL: interleukin; ILC2: type 2 innate lymphoid cell; SCF: stem cell factor; TCR: T-cell receptor; TGF-β: transforming growth factor β; Th: T helper cells; TNF-α: tumor necrosis factor α; T reg: regulatory T-cells; TSLP: thymic stromal lymphopoietin; VCAM-1: vascular cell adhesion protein 1.

Our review provides an overview of the atopic march paradigm, and it summarizes the recent knowledge concerning the main factors (genetic and environmental) involved in the etiopathogenesis of atopic-march-related diseases. In addition, different therapeutic approaches to manage allergic diseases were also developed, with a particular focus on MI strategy with the immune-factor-based formulation of 2LALERG^®^. The therapeutic potential of this medicine, according to its specific composition, was widely discussed systematically and completely, as each active substance composing the medicine was contextualized in relation to the different atopic-march-related diseases. In complement to the text, we have provided a schematic and recapitulative picture of the immunological mechanisms underlying the atopic march, focusing on the main roles played by each cytokine and immune factor used in formulating 2LALERG^®^ (Figure 4). In addition, the current preclinical and clinical evidence concerning 2LALERG^®^ has been reported and discussed. To date, investigations have only focused on the effects of the medicine in the context of pollen-mediated RA, but there is greater potential to be explored, which needs further assessment. The multi-target strategy employed by this MI medicine could help reduce IgE-mediated inflammation, histamine release, and typical symptoms of allergic reactions. In parallel, although the atopic march theory has contributed to the elucidation of the onset of allergic-mediated diseases, many issues remain to be explained and discovered that could help manage the diseases and improve patients’ quality of life. Additional large, epidemiologic studies and clinical trials are needed to evaluate treatment efficacy with respect to each patient’s identity. Ideally, such studies should be set up with improved methods for data collection that consider disease heterogeneity, genetic and environmental aspects, and the eventual presence of comorbid disorders. In addition, in the era of precision medicine, a global vision of the clinical history, including the prescribed drugs, as well as the patients’ self-medication(s), should also be taken into consideration in future research. Since real-life observational studies use recent methods such as mobile applications [153], it would be of great interest to take advantage of these technologies to evaluate the impact of MI as another therapeutic option in this context.

## Figures and Tables

**Table 1:** Composition of 2LALERG^®^.

MIM Composition
hr-IL-1β (17 CH)
hr-IL-4 (17–27 CH)
hr-IL-5 (17 CH)
hr-IL-6 (17 CH)
hr-IL-10 (17 CH)
hr-IL-12 (9 CH)
hr-IL-13 (17 CH)
hr-TNF-α (17 CH)
hr-TGF-β (5 CH)
*Pulmo-histaminum* (15 CH)
SNA^®^-HLA II (18 CH)

CH: centesimal Hahnemannian; HLA: human leucocyte antigen; hr: human recombinant; IL: interleukin; MIM: micro-immunotherapy medicine; SNA^®^: specific nucleic acid; TGF-β: transforming growth factor β; TNF-α: tumor necrosis factor α.

## Abbreviations

AA: allergic asthma; AAD: allergic airway diseases; ACQ-5: 5-item asthma control questionnaire; AD: atopic dermatitis; AHR: airway hyperresponsiveness; AR: allergic rhinitis; ASIT: allergen-specific immunotherapy; BAL: bronchoalveolar lavages; BALF: bronchoalveolar lavages fluids; C5a: complement factor 5a; CH: centesimal Hahnemannian; CLA: cutaneous lymphocyte-associated antigen; CNTF: ciliary neurotropic factor; DC: dendritic cells; Co-Smad: common Smad; CRP: C-reactive protein; DEFB1: human β-defensin-1; DPP-4: dipeptidyl peptidase-4; EAACI: European academy of allergology and clinical immunology; EASI: eczema area and severity index; EGPA: eosinophilic granulomatosis with polyangiitis; ERK 1/2: extra-cellular signal-regulated kinase 1/2; FCER1A: Fc fragment of IgE receptor Ia; FEV_1_: forced expiratory volume in 1 s; FMLP: N-formyl-methionyl-leucyl-phenylalanine; FVB: Friend leukemia virus B; GM-CSF: Granulocyte-Macrophage Colony-Stimulating Factor; HBD-3: human-β defensin-3; HDM: house-dust mite; HLA: human leukocyte antigen; IFN-γ: γ interferon; Ig: immunoglobulin; IL: interleukin; IL-1Ra: IL-1 antagonist receptor; IL-4R: IL-4 receptor; ILC2: type 2 innate lymphoid cell; ISAAC: international study of asthma and allergies in childhood; I-Smads: inhibitory Smads; JNK: c-Jun N-terminal kinase; LAP: latency associated protein; LD: low doses; LPS: lipopolysaccharide; LTBP: latent-TGF-β-protein; LTC4: leukotriene C4; MAPK mitogen-activated protein kinase; MHC: major histocompatibility complex; MI: micro-immunotherapy; MIM: micro-immunotherapy medicine; MSE: maternal smoking exposure; NAP-1: nucleosome assembly protein 1; NK: natural killer cell; OVA: ovalbumin; pAPC: professional antigen presenting cells; PBMC: peripheral blood mononuclear cell; PBS: phosphate-buffered saline; PGD2: prostaglandin D2; PI3K/AKT: phosphatidylinositol 3-kinase/Ak strain transforming; r-NAP-1: recombinant neutrophil-activating peptide 1; R-Smads: receptor-associated Smads; SCF: stem cell factor; SCORAD: SCORing Atopic Dermatitis; SGRQ: St. George’s respiratory questionnaire; slanMo: 6-sulfo LacNAc-expressing monocytes; SNA^®^: specific nucleic acid; T5SS: total 5 symptoms score; TARC: thymus and activation regulated chemokine; TCR: T-cell receptor; TDI: toluene-2,4-diisocyanate; TGF-β: transforming growth factor β; Th2: T helper 2; TNF-α: tumor necrosis factor α; TNFRSF6B: TNF receptor superfamily member 6b; Treg: regulatory T-cells; TSLP: thymic stromal lymphopoietin; ULD: ultra-low doses; VCAM-1: vascular cell adhesion protein 1.
